# Supplementary material for: A Humanized Yeast Phenomic Model of Deoxycytidine Kinase to Predict Genetic Buffering of Nucleoside Analog Cytotoxicity
Source: Genes (Basel). 2019 Sep 30;10(10):770. doi: 10.3390/genes10100770 (PMC6826991; doi:10.3390/genes10100770)
Supplement: Supplementary file 1 [file genes-10-00770-s001.zip › Additional_Files/Additional_File7_GOTermHeatmaps.zip!/Additional_File7_GOTermHeatmaps/RNA_binding.pdf]

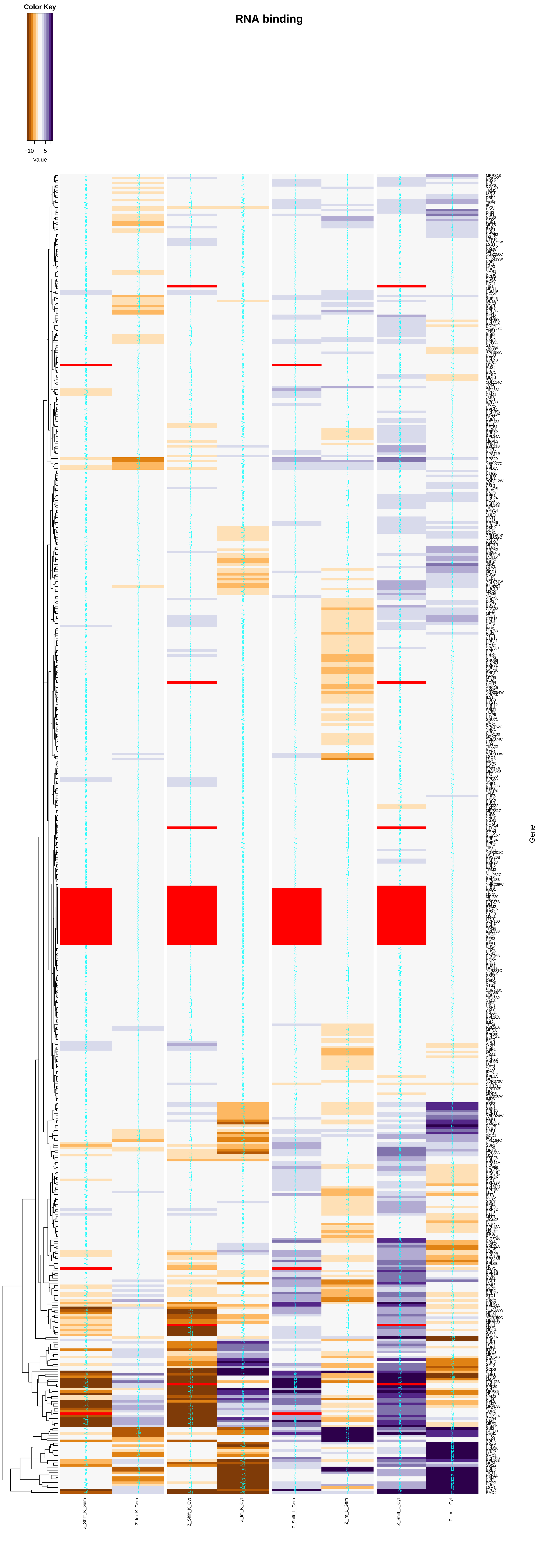
Color Key
RNA binding
−10
5
Value
Gene
Z_Shift_K_Gem
Z_Im_K_Gem
Z_Shift_K_Cyt
Z_Im_K_Cyt
Z_Shift_L_Gem
Z_Im_L_Gem
Z_Shift_L_Cyt
Z_Im_L_Cyt

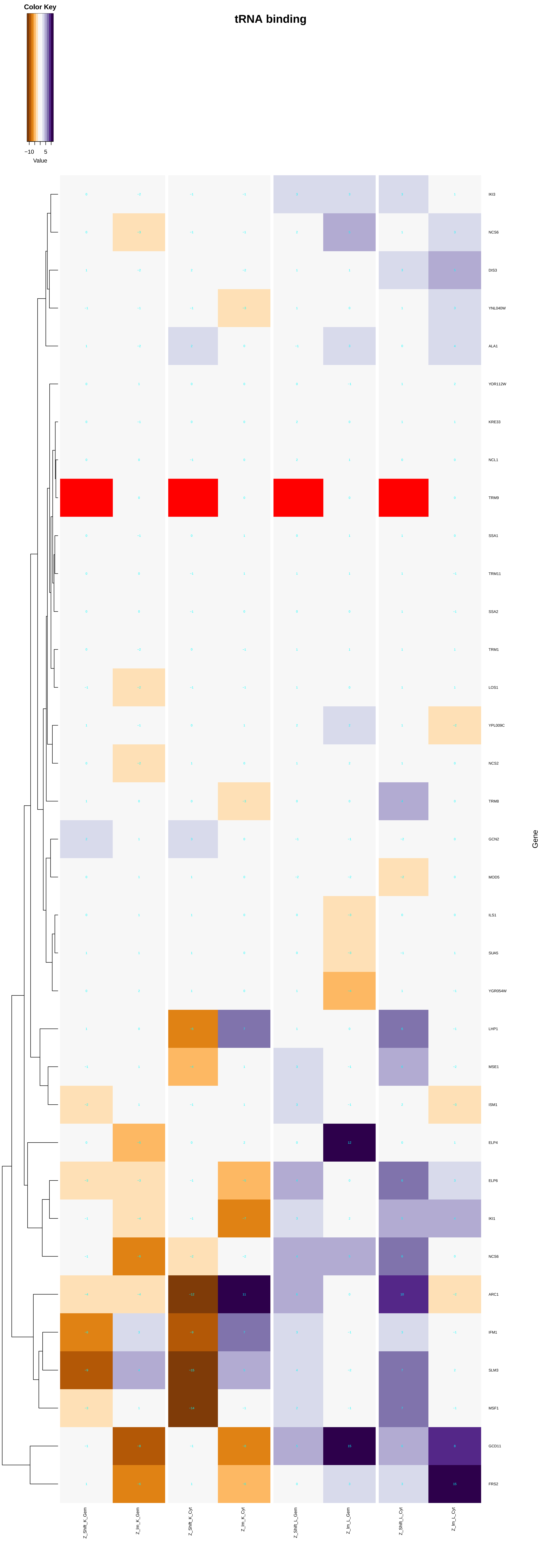
tRNA binding
Color Key
−10 5
Value
IKI3
NCS6
DIS3
YNL040W
ALA1
YOR112W
KRE33
NCL1
TRM9
SSA1
TRM11
SSA2
TRM1
LOS1
YPL009C
NCS2
TRM8
GCN2
MOD5
ILS1
SUA5
YGR054W
LHP1
MSE1
ISM1
ELP4
ELP6
IKI1
NCS6
ARC1
IFM1
SLM3
MSF1
GCD11
FRS2
Gene
Z_Shift_K_Gem
Z_Im_K_Gem
Z_Shift_K_Cyt
Z_Im_K_Cyt
Z_Shift_L_Gem
Z_Im_L_Gem
Z_Shift_L_Cyt
Z_Im_L_Cyt

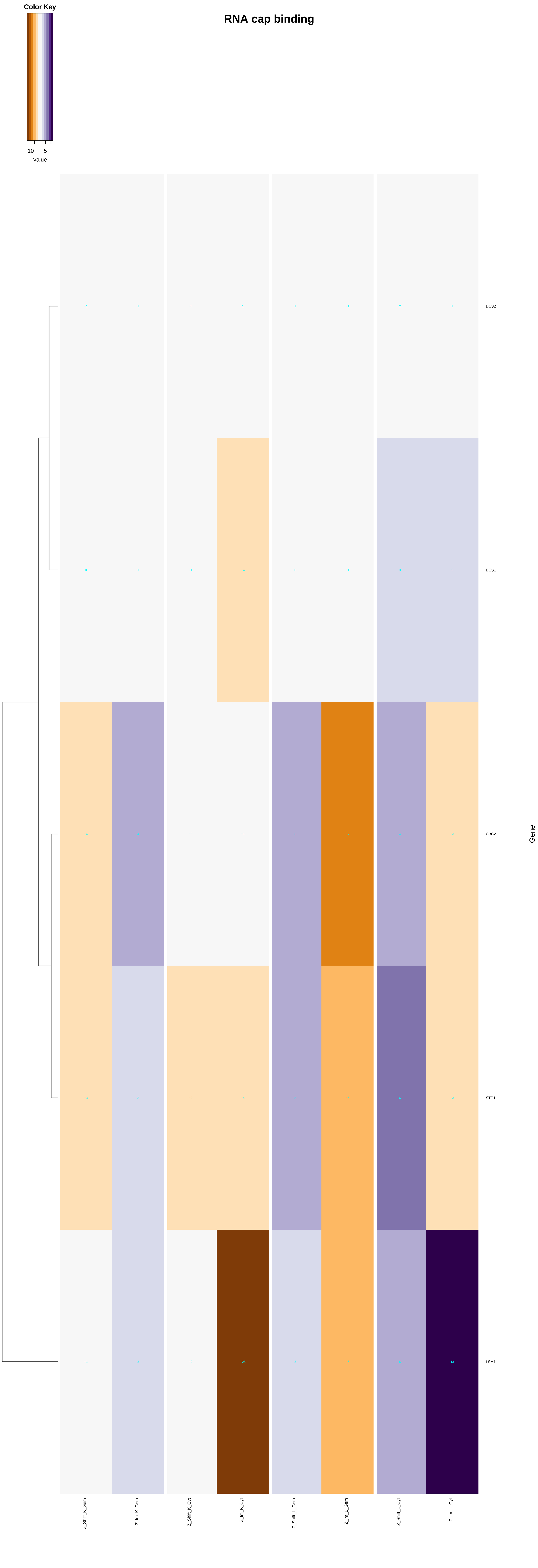

Color Key
−10
5
Value
RNA cap binding
DCS2
DCS1
CBC2
STO1
LSM1
Gene
Z_Shift_K_Gem
Z_Im_K_Gem
Z_Shift_K_Cyt
Z_Im_K_Cyt
Z_Shift_L_Gem
Z_Im_L_Gem
Z_Shift_L_Cyt
Z_Im_L_Cyt

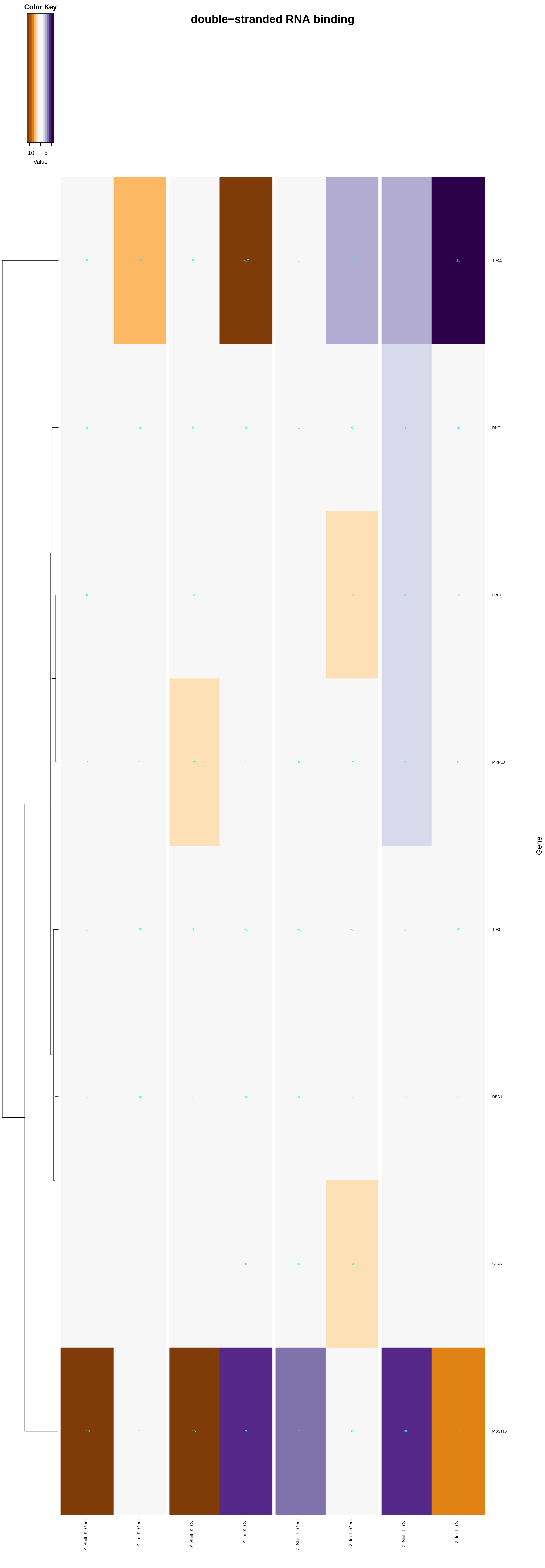
double−stranded RNA binding
Color Key
−10
5
Value
TIF11
RNT1
LRP1
MRPL3
TIF3
DED1
SUA5
MSS116
Gene
Z_Shift_K_Gem
Z_Im_K_Gem
Z_Shift_K_Cyt
Z_Im_K_Cyt
Z_Shift_L_Gem
Z_Im_L_Gem
Z_Shift_L_Cyt
Z_Im_L_Cyt

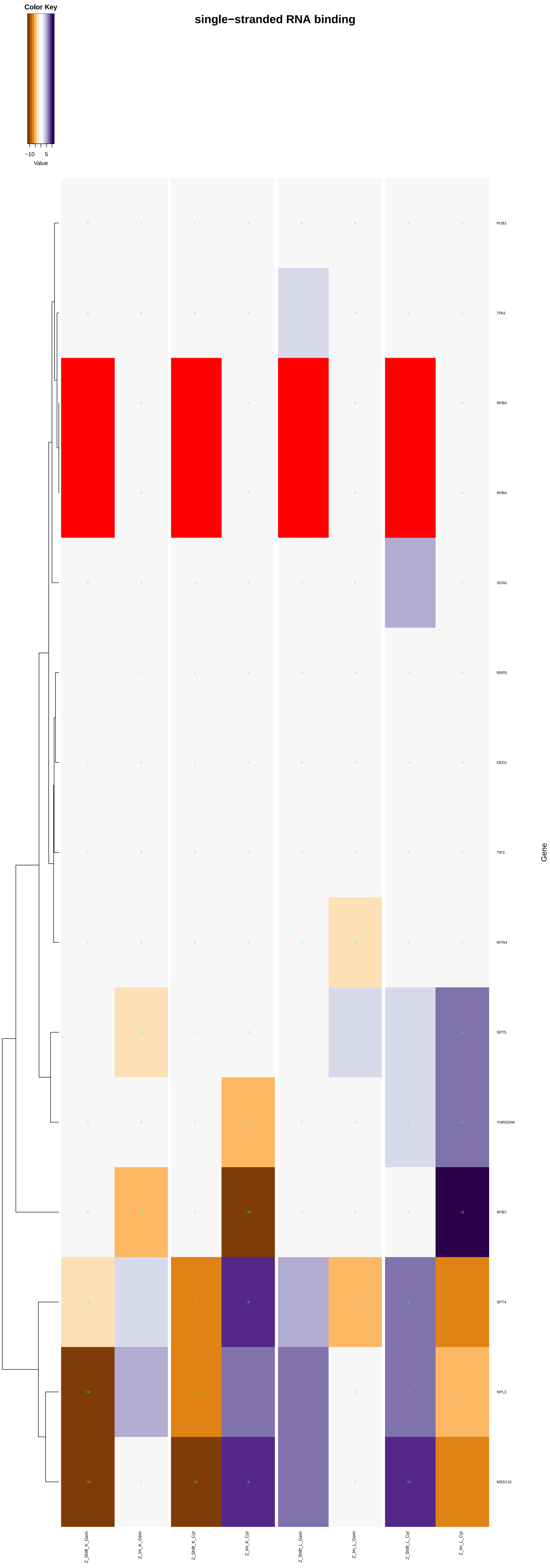
Color Key
−10
5
Value
single−stranded RNA binding
PUB1
TPA1
RPB4
RPB4
SGN1
RRP5
DED1
TIF3
MTR4
SPT5
YNR024W
RPB7
SPT4
NPL3
MSS116
Gene
Z_Shift_K_Gem
Z_Im_K_Gem
Z_Shift_K_Cyt
Z_Im_K_Cyt
Z_Shift_L_Gem
Z_Im_L_Gem
Z_Shift_L_Cyt
Z_Im_L_Cyt

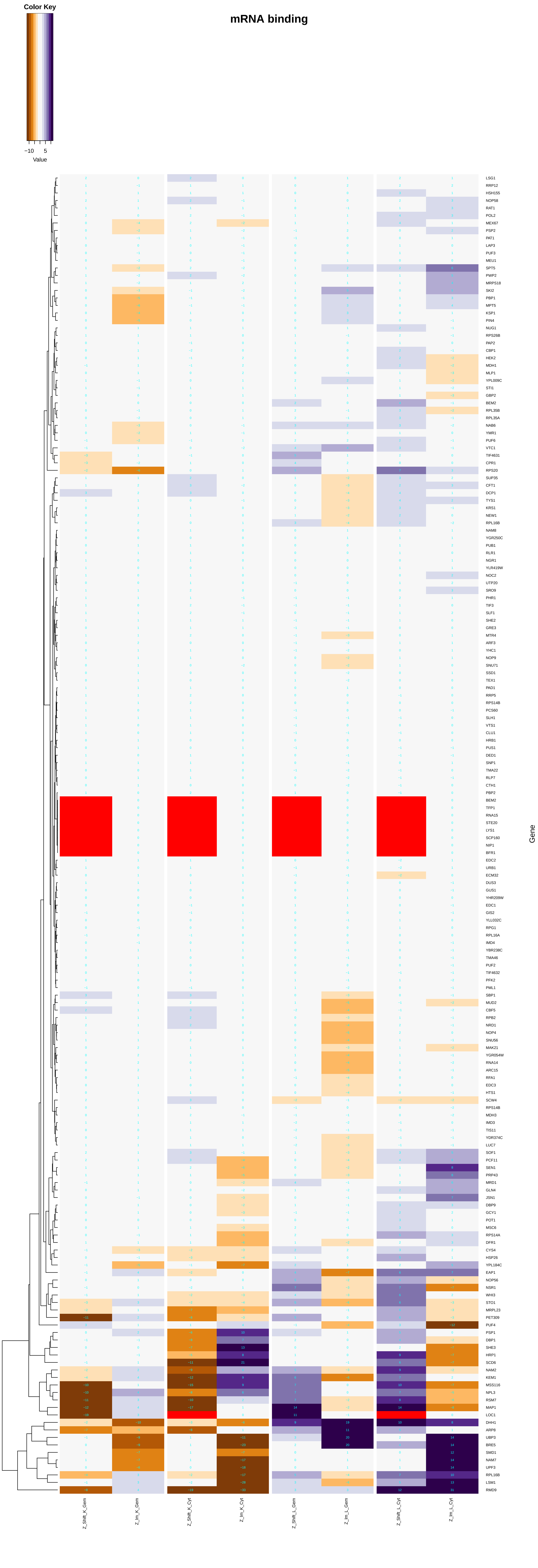

mRNA binding
Color Key
−10
5
Value
Gene
Z_Shift_K_Gem
Z_Im_K_Gem
Z_Shift_K_Cyt
Z_Im_K_Cyt
Z_Shift_L_Gem
Z_Im_L_Gem
Z_Shift_L_Cyt
Z_Im_L_Cyt

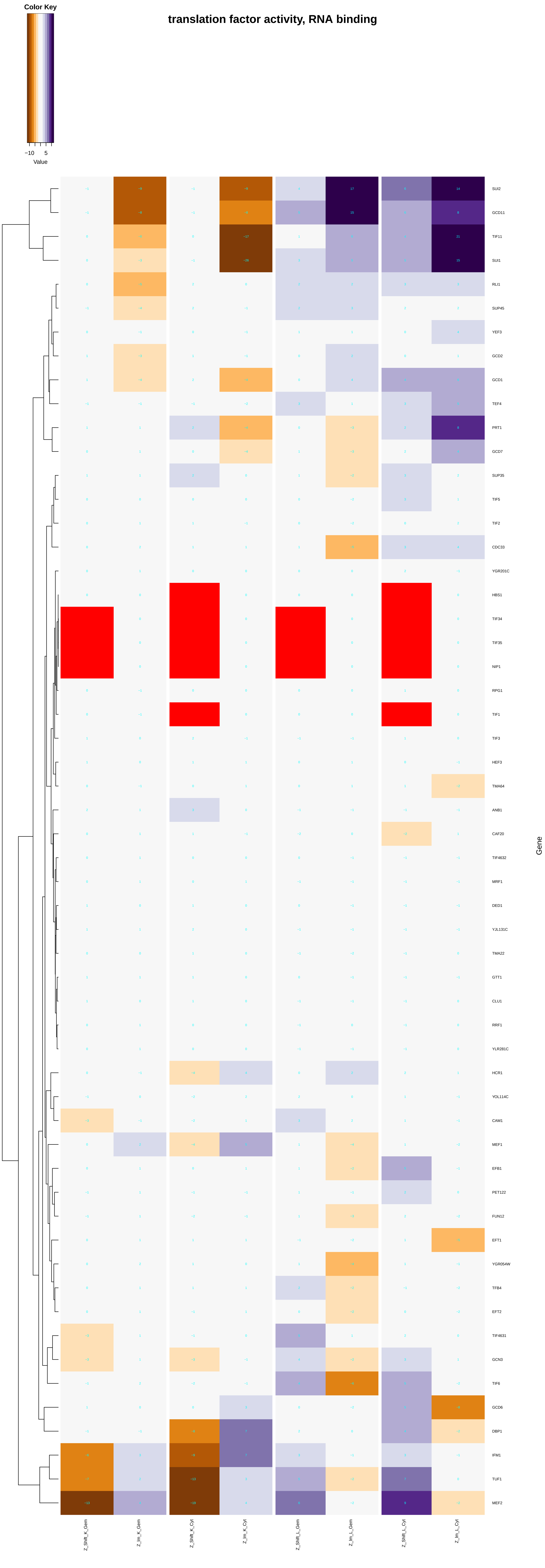
translation factor activity, RNA binding
Color Key
−10
5
Value
SUI2
GCD11
TIF11
SUI1
RLI1
SUP45
YEF3
GCD2
GCD1
TEF4
PRT1
GCD7
SUP35
TIF5
TIF2
CDC33
YGR201C
HBS1
TIF34
TIF35
NIP1
RPG1
TIF1
TIF3
HEF3
TMA64
ANB1
CAF20
TIF4632
MRF1
DED1
YJL131C
TMA22
GTT1
CLU1
RRF1
YLR281C
HCR1
YOL114C
CAM1
MEF1
EFB1
PET122
FUN12
EFT1
YGR054W
TFB4
EFT2
TIF4631
GCN3
TIF6
GCD6
DBP1
IFM1
TUF1
MEF2
Gene
Z_Shift_K_Gem
Z_Im_K_Gem
Z_Shift_K_Cyt
Z_Im_K_Cyt
Z_Shift_L_Gem
Z_Im_L_Gem
Z_Shift_L_Cyt
Z_Im_L_Cyt

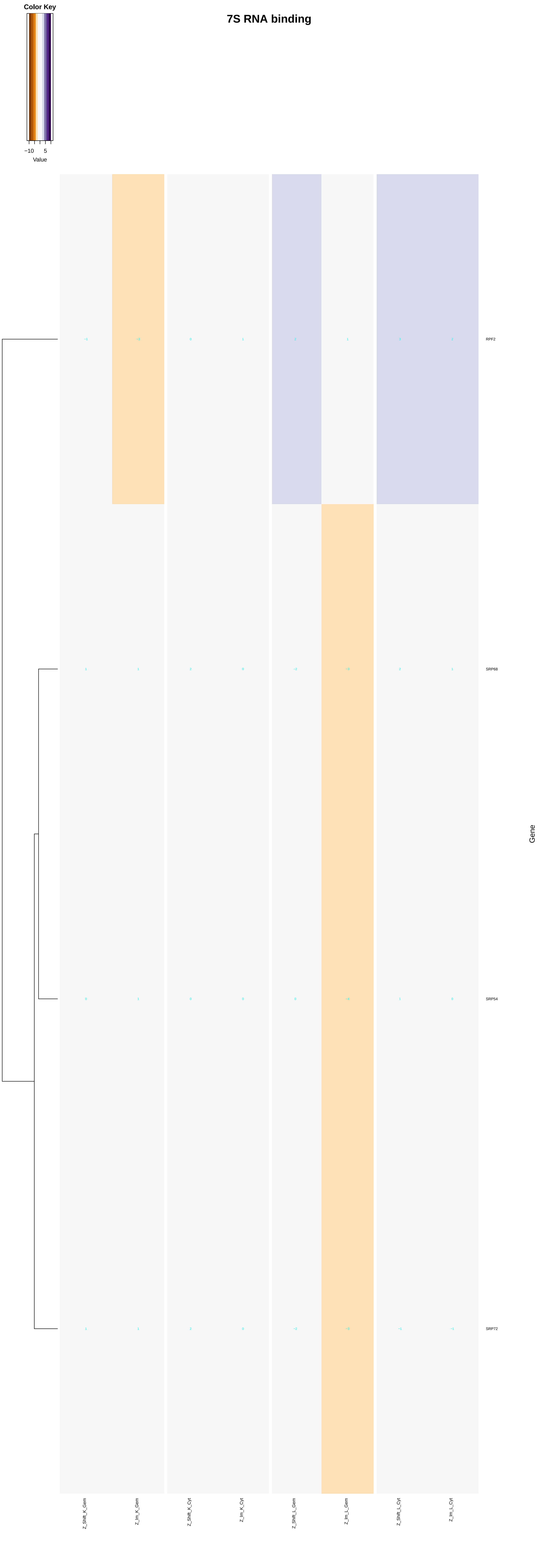

7S RNA binding
Color Key
−10
5
Value
RPF2
SRP68
SRP54
SRP72
Gene
Z_Shift_K_Gem
Z_Im_K_Gem
Z_Shift_K_Cyt
Z_Im_K_Cyt
Z_Shift_L_Gem
Z_Im_L_Gem
Z_Shift_L_Cyt
Z_Im_L_Cyt

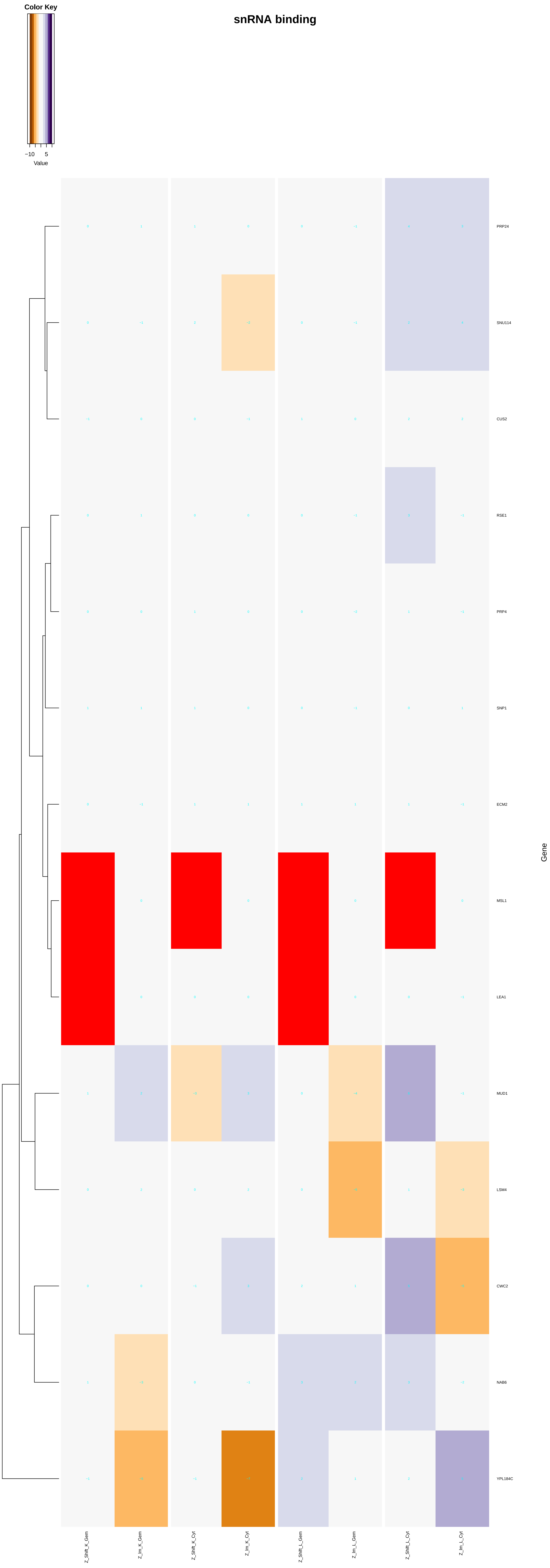
Color Key
−10
5
Value
snRNA binding
PRP24
SNU114
CUS2
RSE1
PRP4
SNP1
ECM2
MSL1
LEA1
MUD1
LSM4
CWC2
NAB6
YPL184C
Gene
Z_Shift_K_Gem
Z_Im_K_Gem
Z_Shift_K_Cyt
Z_Im_K_Cyt
Z_Shift_L_Gem
Z_Im_L_Gem
Z_Shift_L_Cyt
Z_Im_L_Cyt

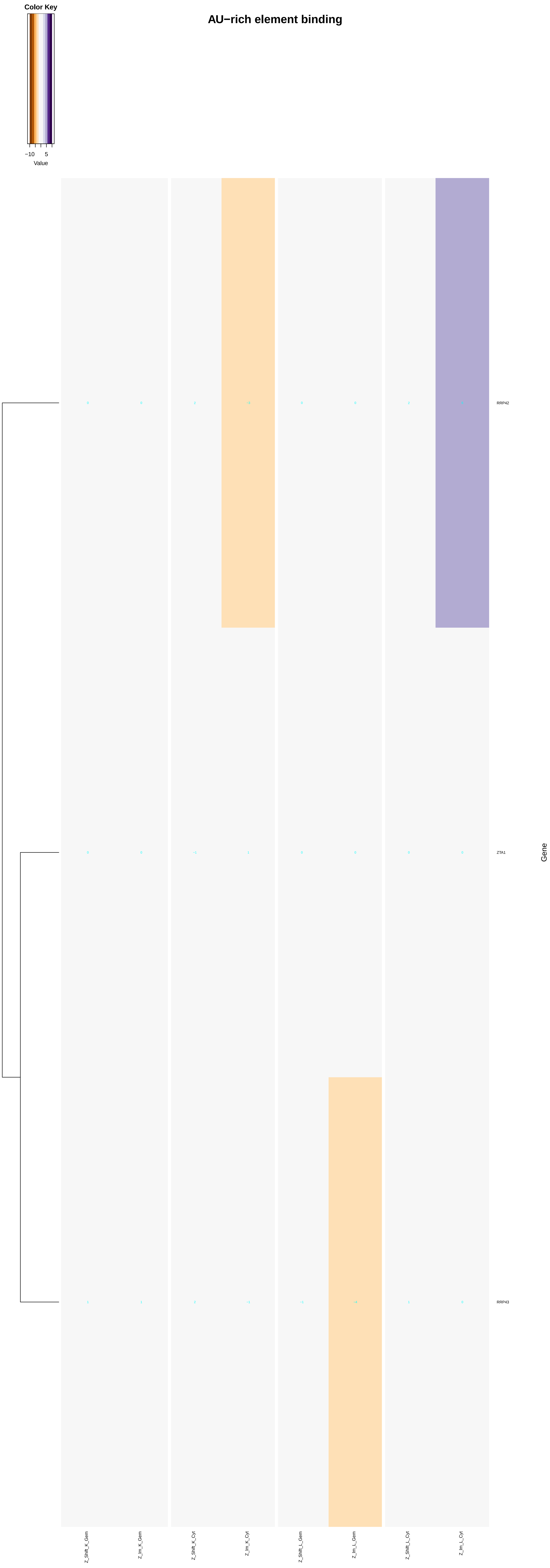

AU−rich element binding
Color Key
−10
5
Value
RRP42
ZTA1
RRP43
Gene
Z_Shift_K_Gem
Z_Im_K_Gem
Z_Shift_K_Cyt
Z_Im_K_Cyt
Z_Shift_L_Gem
Z_Im_L_Gem
Z_Shift_L_Cyt
Z_Im_L_Cyt

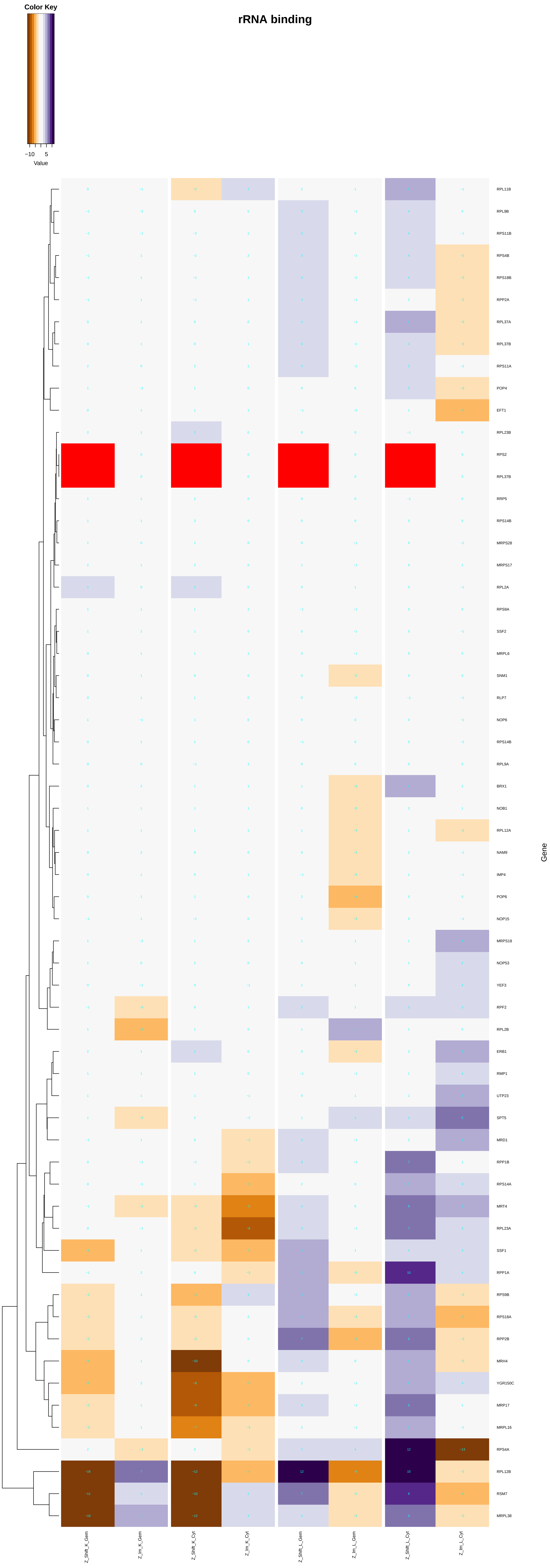
rRNA binding
Color Key
−10
5
Value
RPL11B
RPL9B
RPS11B
RPS4B
RPS18B
RPP2A
RPL37A
RPL37B
RPS11A
POP4
EFT1
RPL23B
RPS2
RPL37B
RRP5
RPS14B
MRPS28
MRPS17
RPL2A
RPS9A
SSF2
MRPL6
SNM1
RLP7
NOP6
RPS14B
RPL9A
BRX1
NOB1
RPL12A
NAM9
IMP4
POP6
NOP15
MRPS18
NOP53
YEF3
RPF2
RPL2B
ERB1
RMP1
UTP23
SPT5
MRD1
RPP1B
RPS14A
MRT4
RPL23A
SSF1
RPP1A
RPS9B
RPS18A
RPP2B
MRH4
YGR150C
MRP17
MRPL16
RPS4A
RPL12B
RSM7
MRPL38
Gene
Z_Shift_K_Gem
Z_Im_K_Gem
Z_Shift_K_Cyt
Z_Im_K_Cyt
Z_Shift_L_Gem
Z_Im_L_Gem
Z_Shift_L_Cyt
Z_Im_L_Cyt

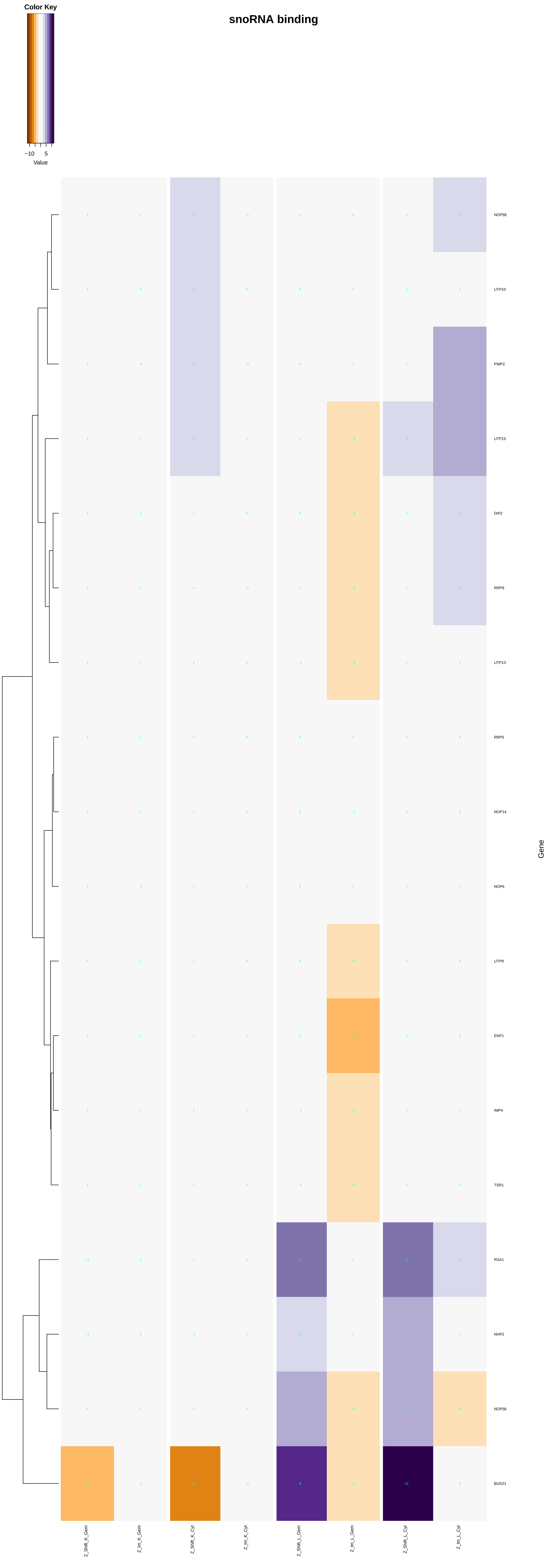
snoRNA binding
Color Key
−10
5
Value
NOP58
UTP10
PWP2
UTP15
DIP2
RRP9
UTP13
RRP5
NOP14
NOP6
UTP9
ENP1
IMP4
TSR1
RSA1
NHP2
NOP56
BUD21
Gene
Z_Shift_K_Gem
Z_Im_K_Gem
Z_Shift_K_Cyt
Z_Im_K_Cyt
Z_Shift_L_Gem
Z_Im_L_Gem
Z_Shift_L_Cyt
Z_Im_L_Cyt

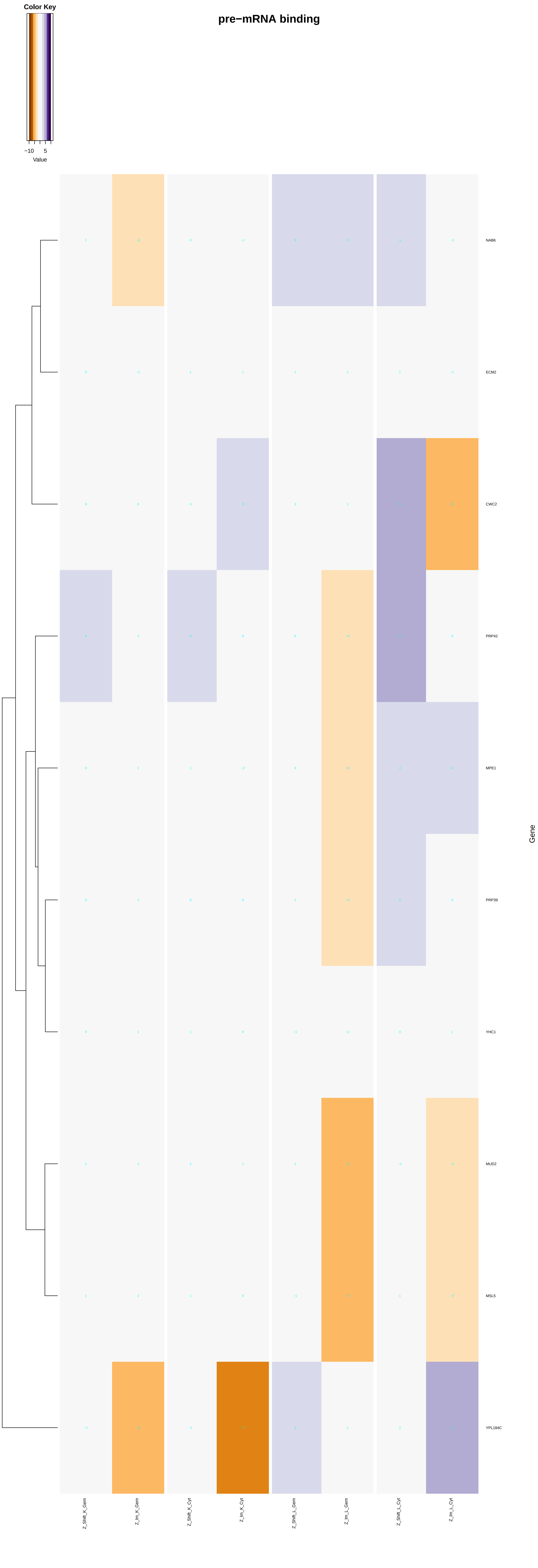

Color Key
−10
5
Value
pre−mRNA binding
NAB6
ECM2
CWC2
PRP42
MPE1
PRP39
YHC1
MUD2
MSL5
YPL184C
Gene
Z_Shift_K_Gem
Z_Im_K_Gem
Z_Shift_K_Cyt
Z_Im_K_Cyt
Z_Shift_L_Gem
Z_Im_L_Gem
Z_Shift_L_Cyt
Z_Im_L_Cyt

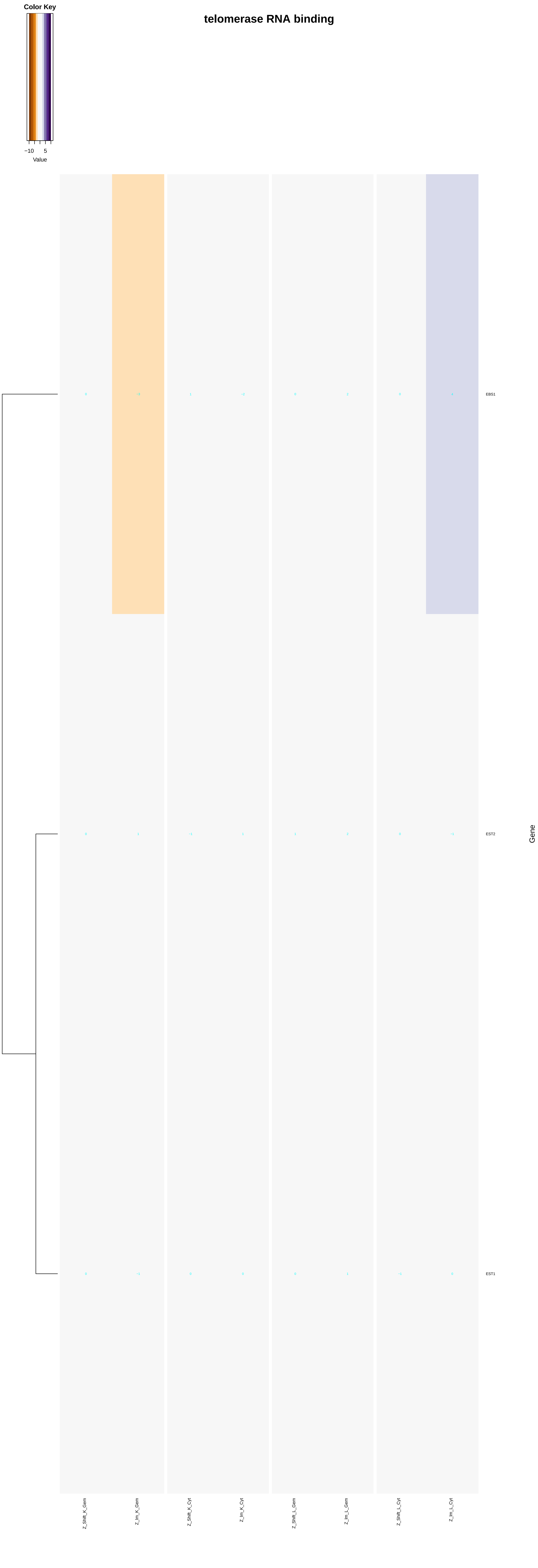

Color Key
−10
5
Value
telomerase RNA binding
EBS1
EST2
EST1
Gene
Z_Shift_K_Gem
Z_Im_K_Gem
Z_Shift_K_Cyt
Z_Im_K_Cyt
Z_Shift_L_Gem
Z_Im_L_Gem
Z_Shift_L_Cyt
Z_Im_L_Cyt

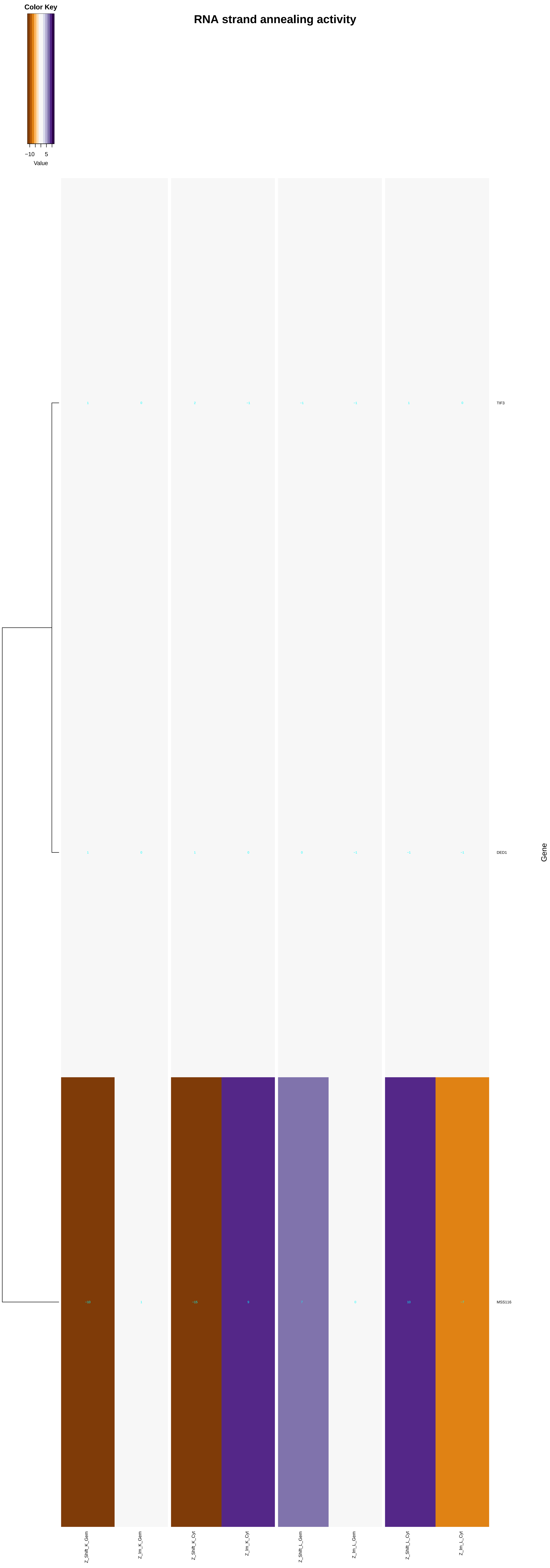
RNA strand annealing activity
Color Key
−10
5
Value
TIF3
DED1
MSS116
Gene
Z_Shift_K_Gem
Z_Im_K_Gem
Z_Shift_K_Cyt
Z_Im_K_Cyt
Z_Shift_L_Gem
Z_Im_L_Gem
Z_Shift_L_Cyt
Z_Im_L_Cyt

poly−pyrimidine tract binding

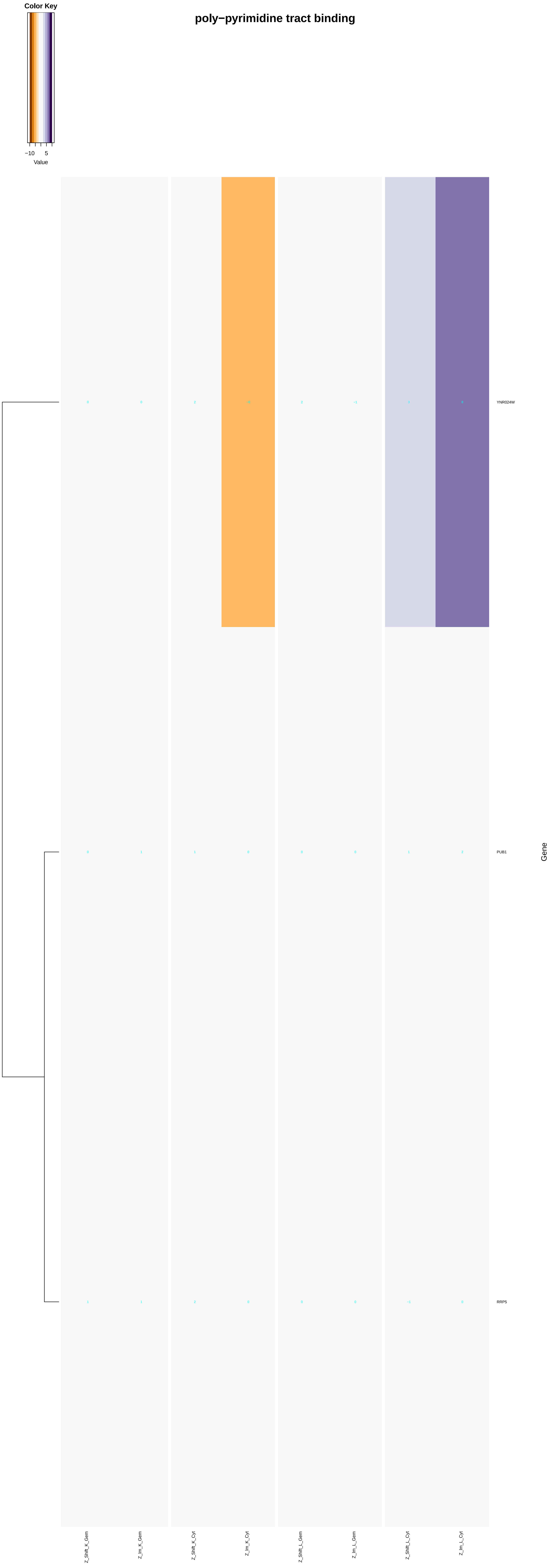

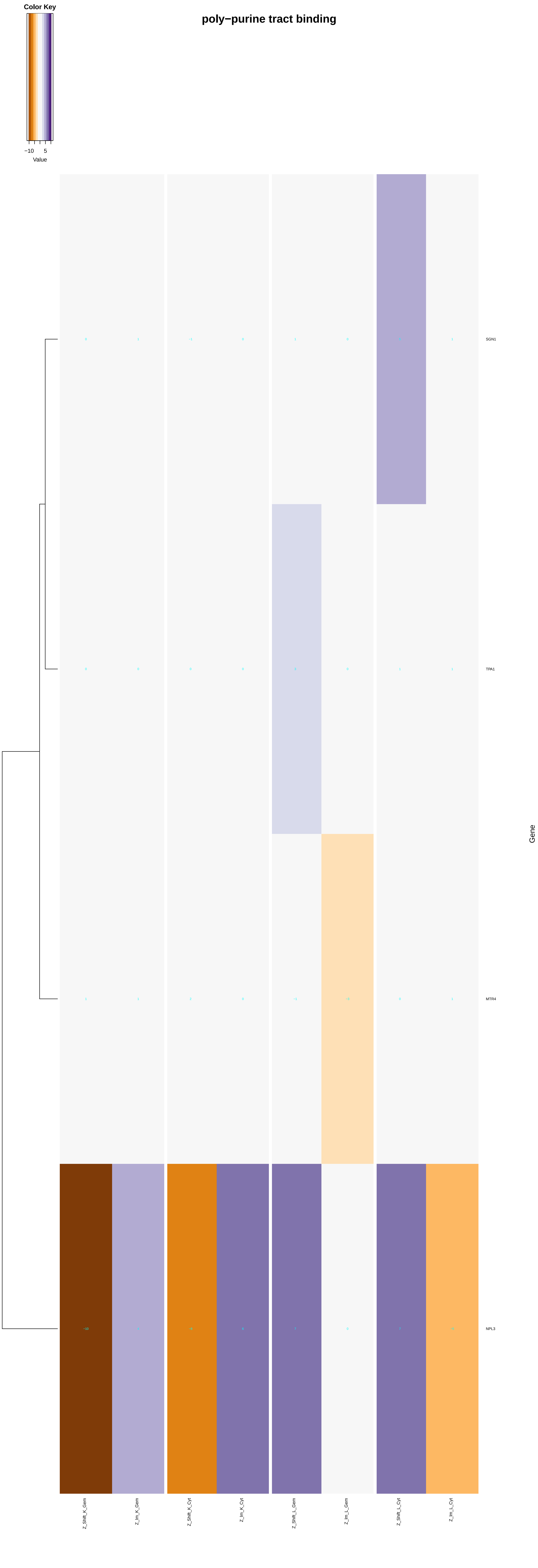
poly−purine tract binding
Color Key
−10
5
Value
SGN1
TPA1
MTR4
NPL3
Gene
Z_Shift_K_Gem
Z_Im_K_Gem
Z_Shift_K_Cyt
Z_Im_K_Cyt
Z_Shift_L_Gem
Z_Im_L_Gem
Z_Shift_L_Cyt
Z_Im_L_Cyt

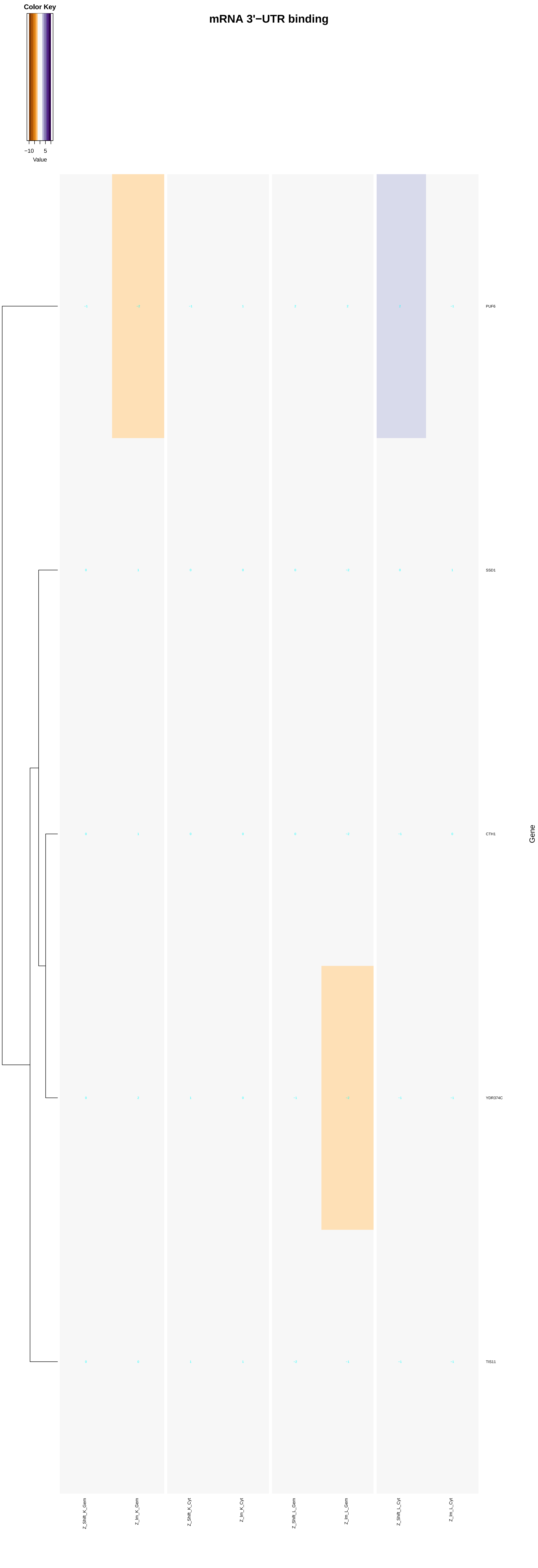

mRNA 3'−UTR binding
Color Key
−10
5
Value
PUF6
SSD1
CTH1
YDR374C
TIS11
Gene
Z_Shift_K_Gem
Z_Im_K_Gem
Z_Shift_K_Cyt
Z_Im_K_Cyt
Z_Shift_L_Gem
Z_Im_L_Gem
Z_Shift_L_Cyt
Z_Im_L_Cyt

mRNA 5'−UTR binding

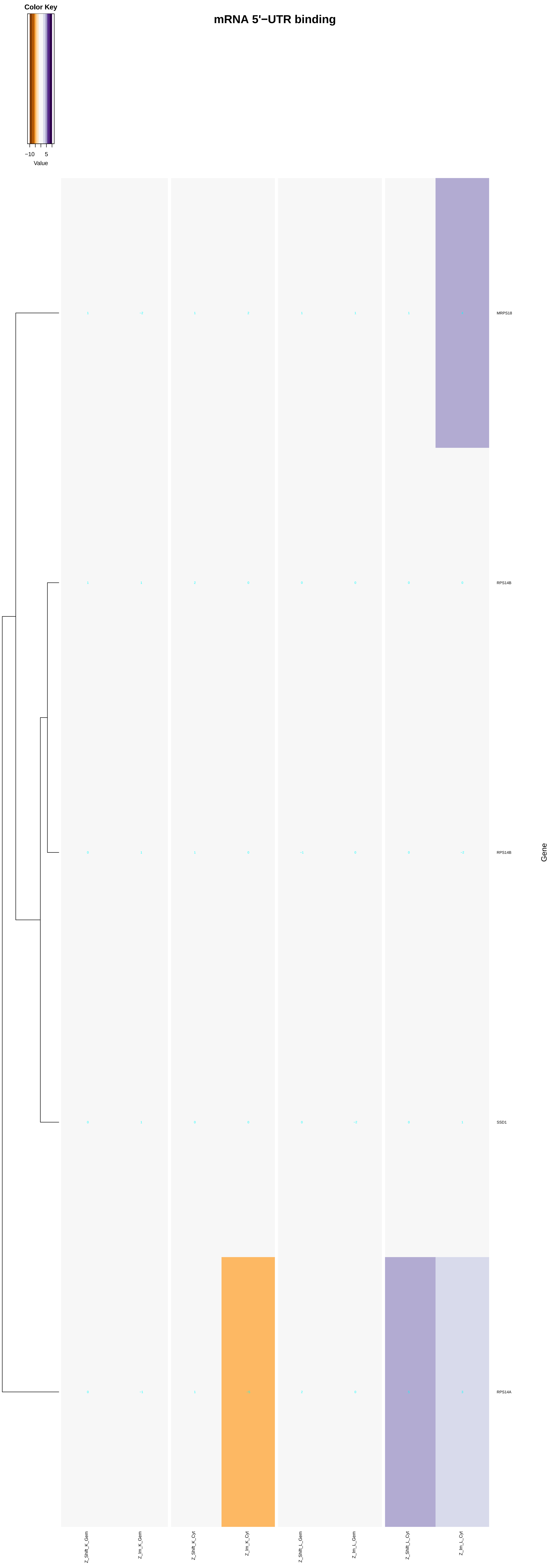

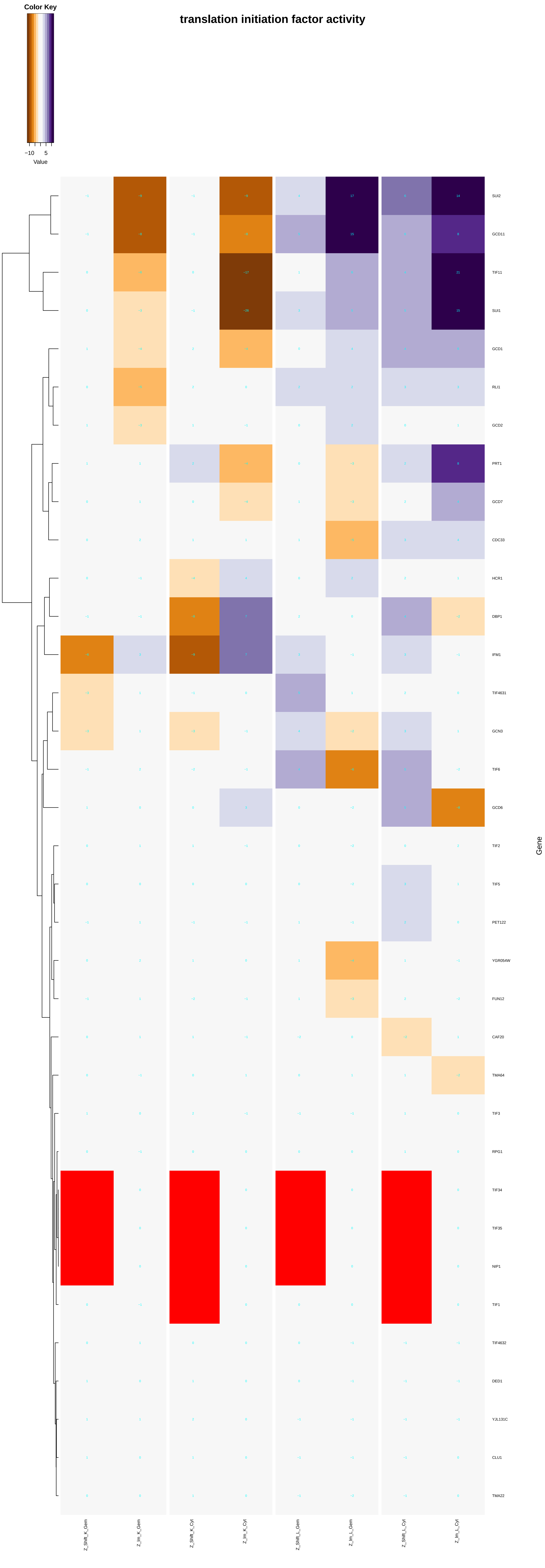
translation initiation factor activity
Color Key
−10
5
Value
SUI2
GCD11
TIF11
SUI1
GCD1
RLI1
GCD2
PRT1
GCD7
CDC33
HCR1
DBP1
IFM1
TIF4631
GCN3
TIF6
GCD6
TIF2
TIF5
PET122
YGR054W
FUN12
CAF20
TMA64
TIF3
RPG1
TIF34
TIF35
NIP1
TIF1
TIF4632
DED1
YJL131C
CLU1
TMA22
Gene
Z_Shift_K_Gem
Z_Im_K_Gem
Z_Shift_K_Cyt
Z_Im_K_Cyt
Z_Shift_L_Gem
Z_Im_L_Gem
Z_Shift_L_Cyt
Z_Im_L_Cyt

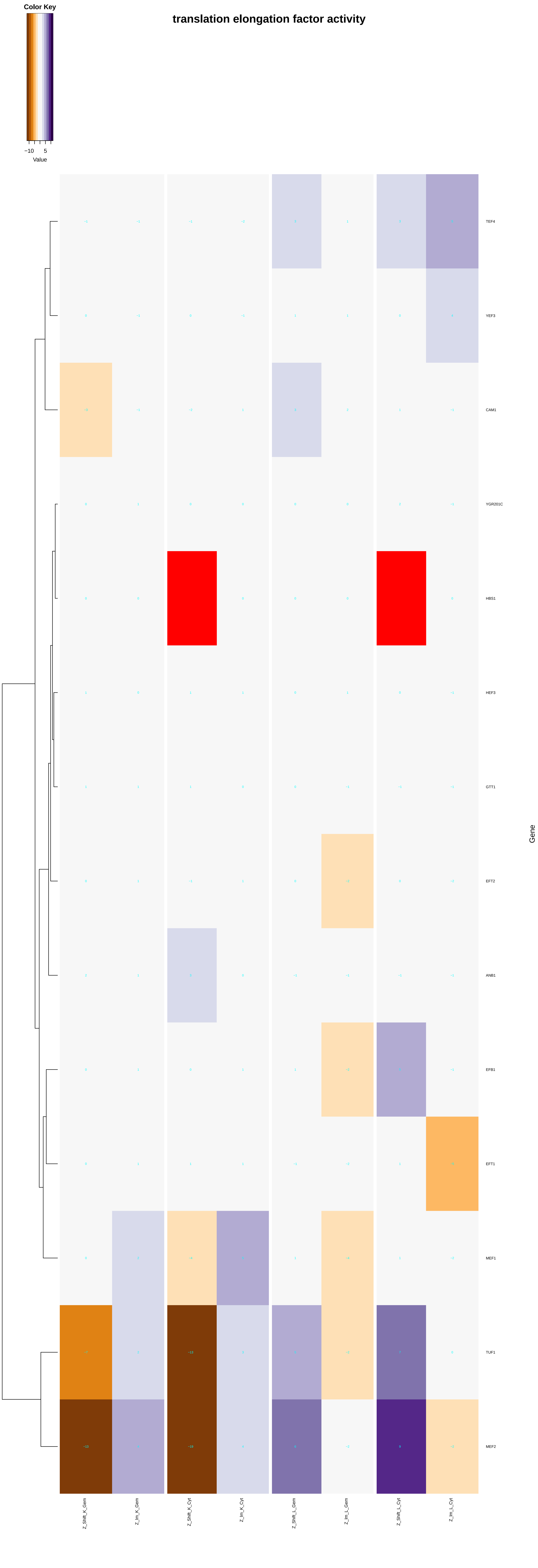

translation elongation factor activity
Color Key
−10
5
Value
TEF4
YEF3
CAM1
YGR201C
HBS1
HEF3
GTT1
EFT2
ANB1
EFB1
EFT1
MEF1
TUF1
MEF2
Gene
Z_Shift_K_Gem
Z_Im_K_Gem
Z_Shift_K_Cyt
Z_Im_K_Cyt
Z_Shift_L_Gem
Z_Im_L_Gem
Z_Shift_L_Cyt
Z_Im_L_Cyt

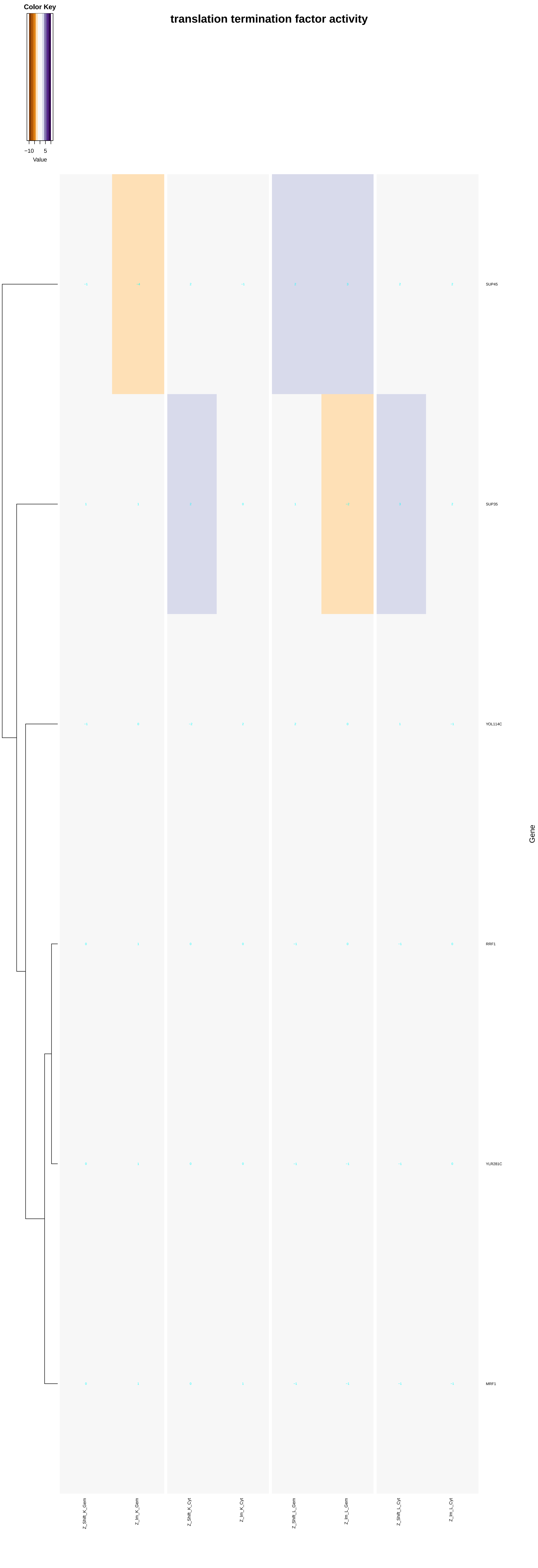
translation termination factor activity
Color Key
−10
5
Value
SUP45
SUP35
YOL114C
RRF1
YLR281C
MRF1
Gene
Z_Shift_K_Gem
Z_Im_K_Gem
Z_Shift_K_Cyt
Z_Im_K_Cyt
Z_Shift_L_Gem
Z_Im_L_Gem
Z_Shift_L_Cyt
Z_Im_L_Cyt

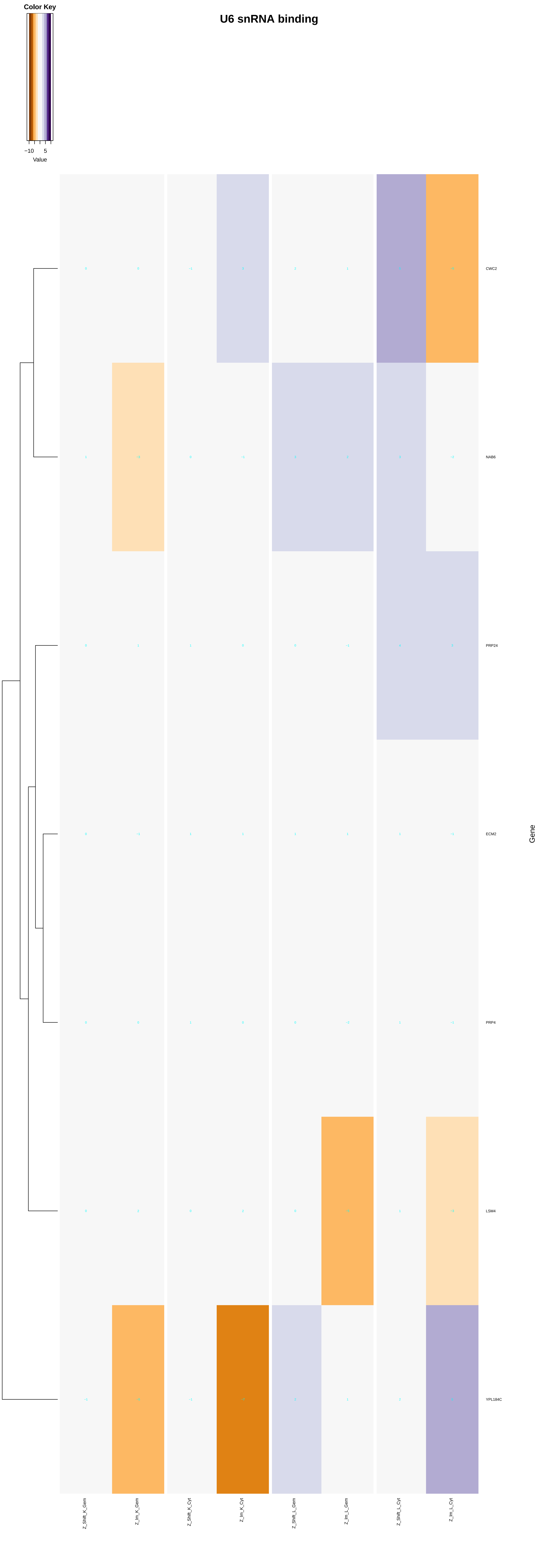
U6 snRNA binding
Color Key
−10
5
Value
CWC2
NAB6
PRP24
ECM2
PRP4
LSM4
YPL184C
Gene
Z_Shift_K_Gem
Z_Im_K_Gem
Z_Shift_K_Cyt
Z_Im_K_Cyt
Z_Shift_L_Gem
Z_Im_L_Gem
Z_Shift_L_Cyt
Z_Im_L_Cyt

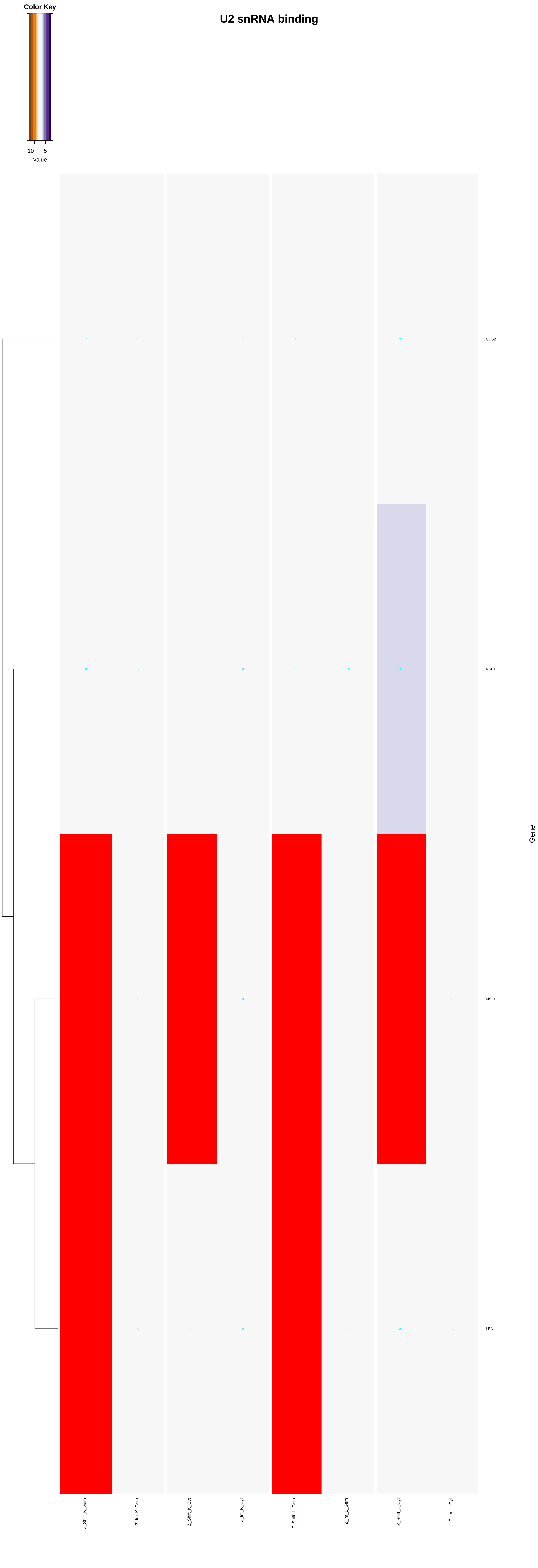
Color Key
−10
5
Value
U2 snRNA binding
CUS2
RSE1
MSL1
LEA1
Gene
Z_Shift_K_Gem
Z_Im_K_Gem
Z_Shift_K_Cyt
Z_Im_K_Cyt
Z_Shift_L_Gem
Z_Im_L_Gem
Z_Shift_L_Cyt
Z_Im_L_Cyt

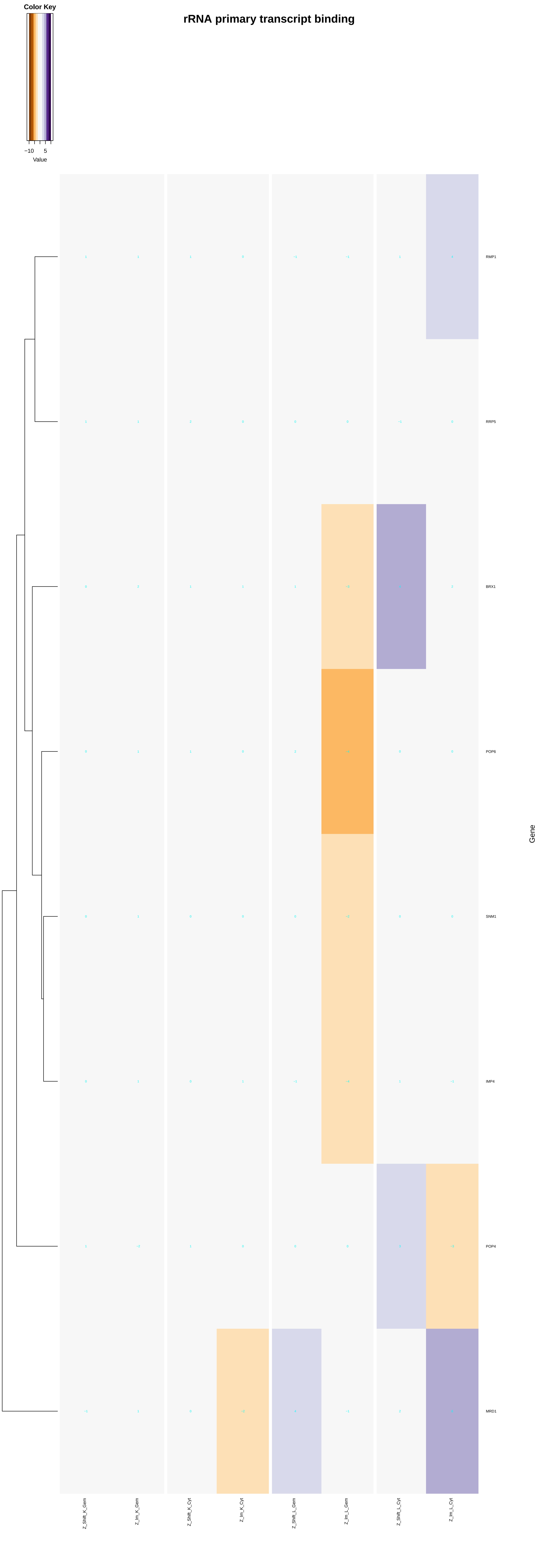
rRNA primary transcript binding
Color Key
−10
5
Value
RMP1
RRP5
BRX1
POP6
SNM1
IMP4
POP4
MRD1
Gene
Z_Shift_K_Gem
Z_Im_K_Gem
Z_Shift_K_Cyt
Z_Im_K_Cyt
Z_Shift_L_Gem
Z_Im_L_Gem
Z_Shift_L_Cyt
Z_Im_L_Cyt

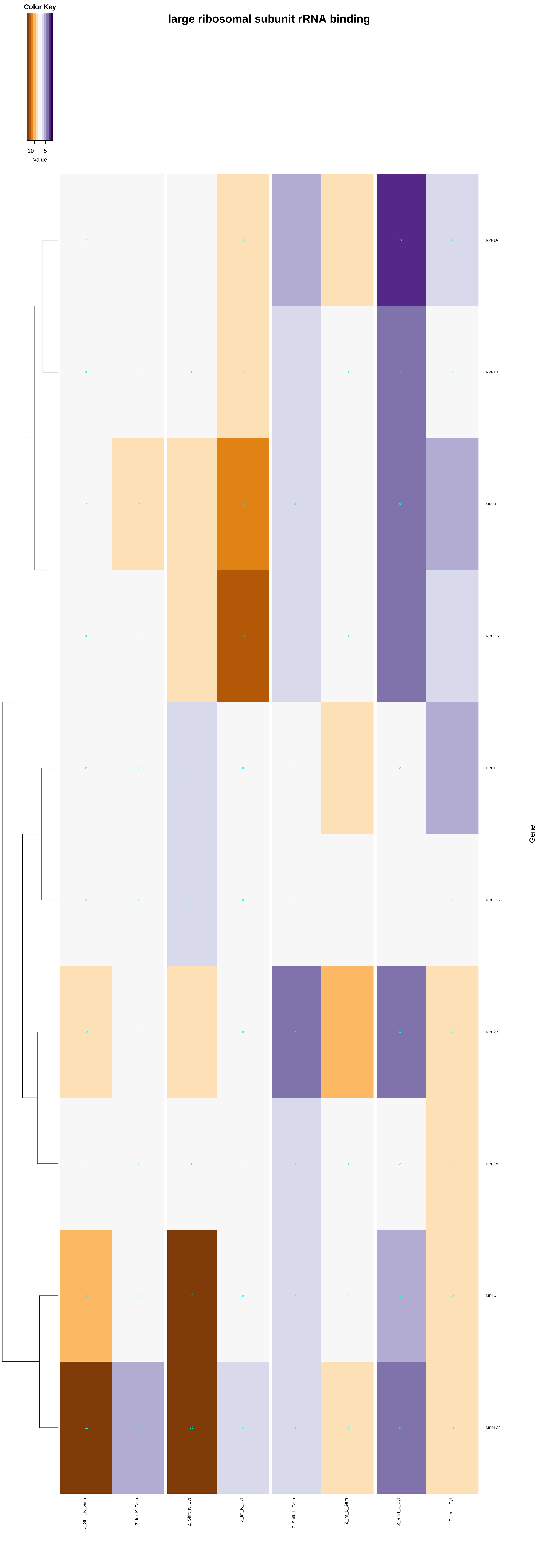
large ribosomal subunit rRNA binding
Color Key
−10
5
Value
RPP1A
RPP1B
MRT4
RPL23A
ERB1
RPL23B
RPP2B
RPP2A
MRH4
MRPL38
Gene
Z_Shift_K_Gem
Z_Im_K_Gem
Z_Shift_K_Cyt
Z_Im_K_Cyt
Z_Shift_L_Gem
Z_Im_L_Gem
Z_Shift_L_Cyt
Z_Im_L_Cyt

small ribosomal subunit rRNA binding

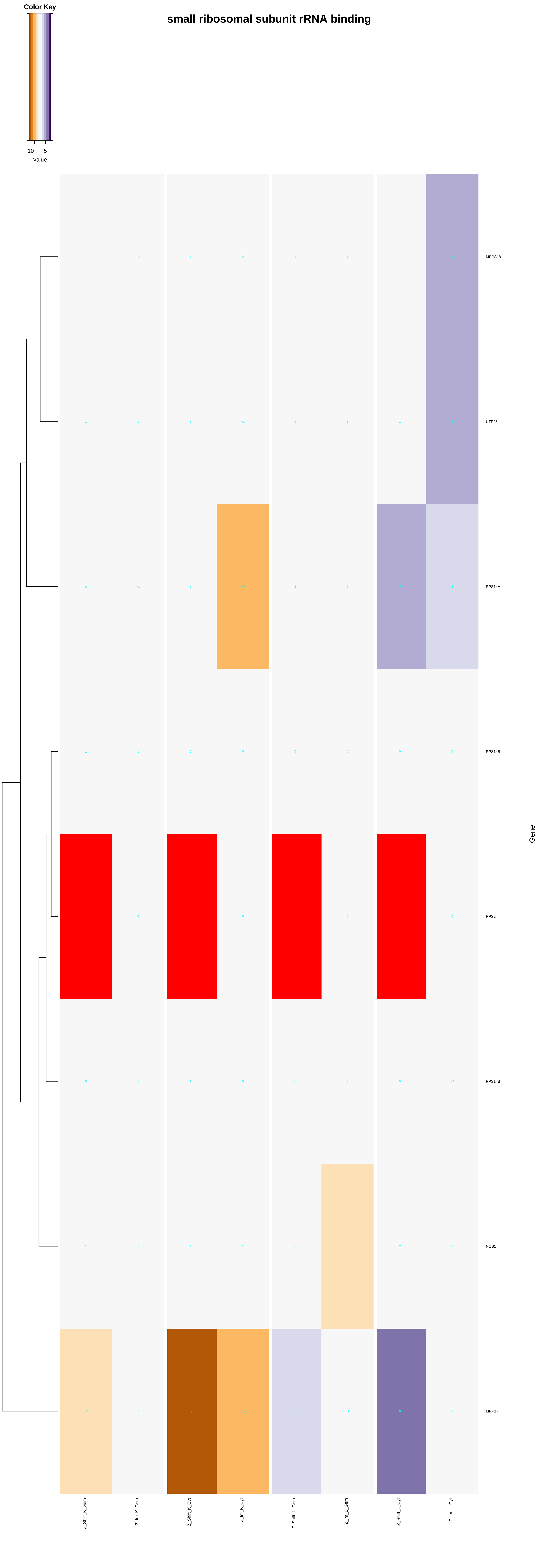

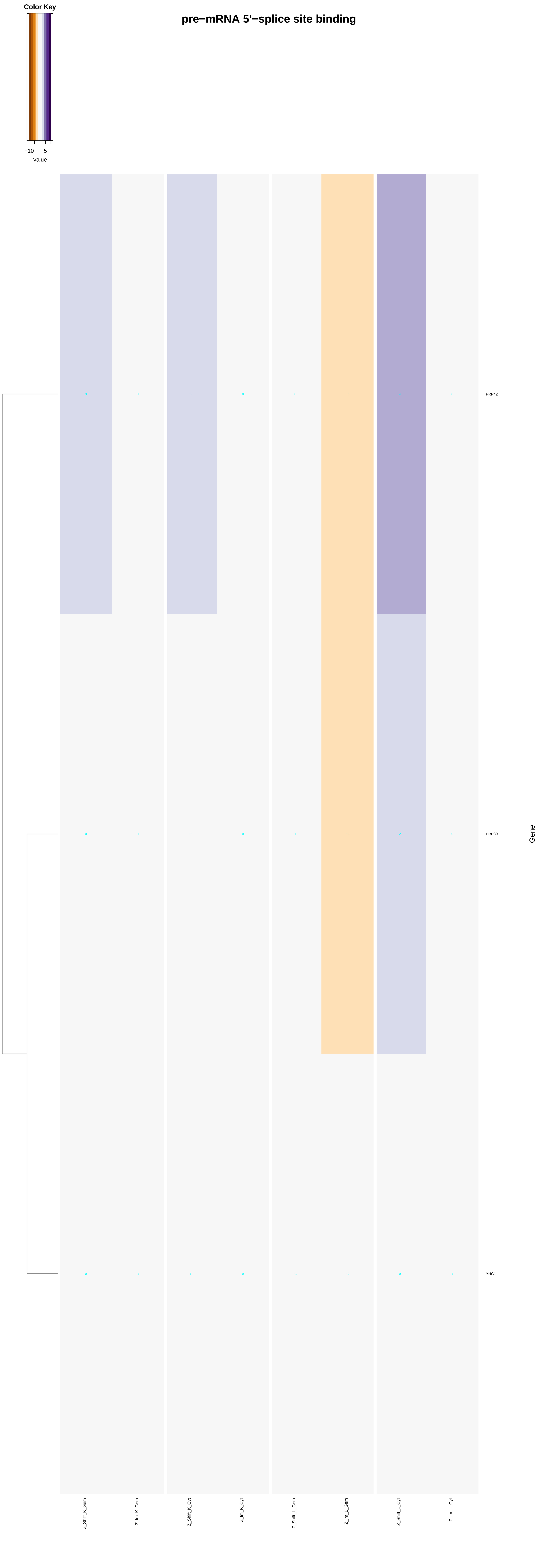
Color Key
−10
5
Value
pre−mRNA 5'−splice site binding
3
1
3
0
0
−3
4
0
PRP42
0
1
0
0
1
−3
2
0
PRP39
0
1
1
0
−1
−2
0
1
YHC1
Gene
Z_Shift_K_Gem
Z_Im_K_Gem
Z_Shift_K_Cyt
Z_Im_K_Cyt
Z_Shift_L_Gem
Z_Im_L_Gem
Z_Shift_L_Cyt
Z_Im_L_Cyt

poly(U) RNA binding

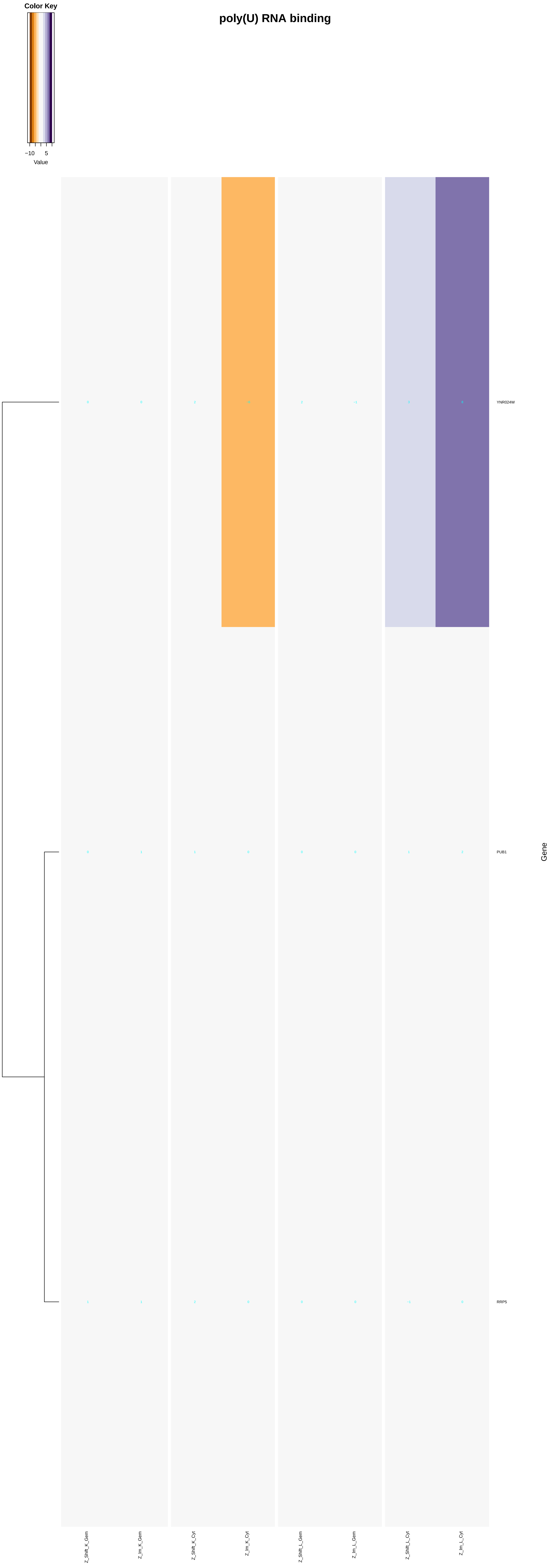

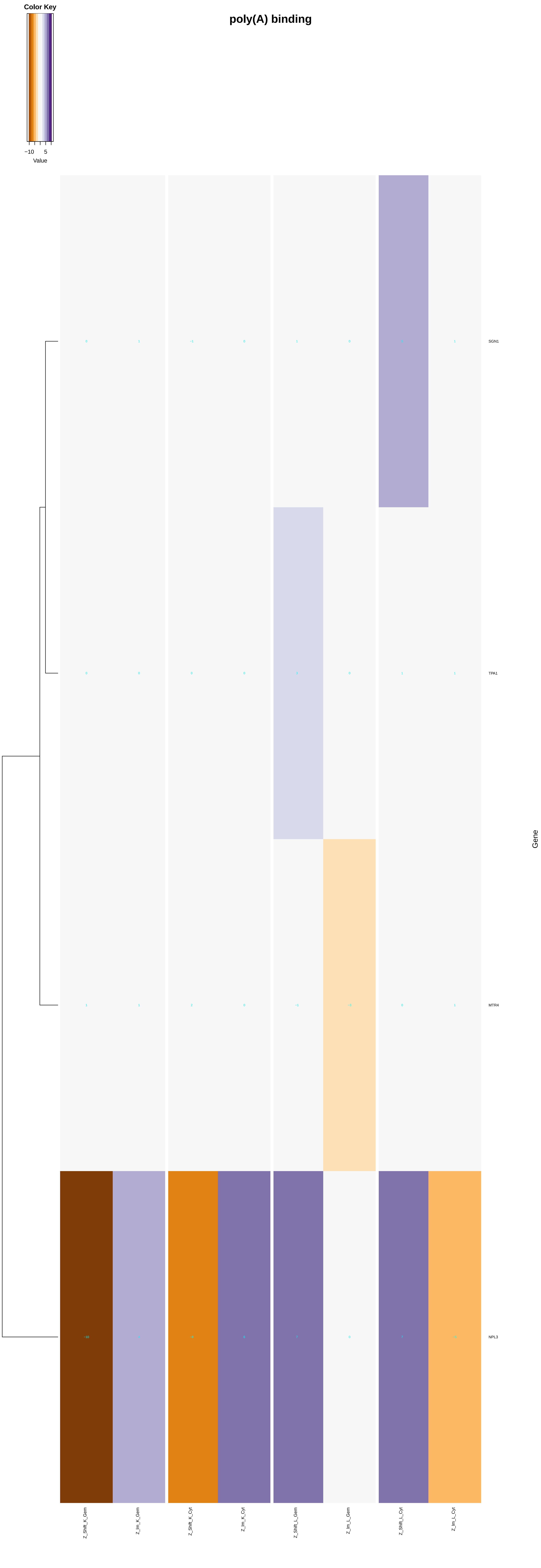

Color Key
−10
5
Value
poly(A) binding
SGN1
TPA1
MTR4
NPL3
Gene
Z_Shift_K_Gem
Z_Im_K_Gem
Z_Shift_K_Cyt
Z_Im_K_Cyt
Z_Shift_L_Gem
Z_Im_L_Gem
Z_Shift_L_Cyt
Z_Im_L_Cyt

translation release factor activity

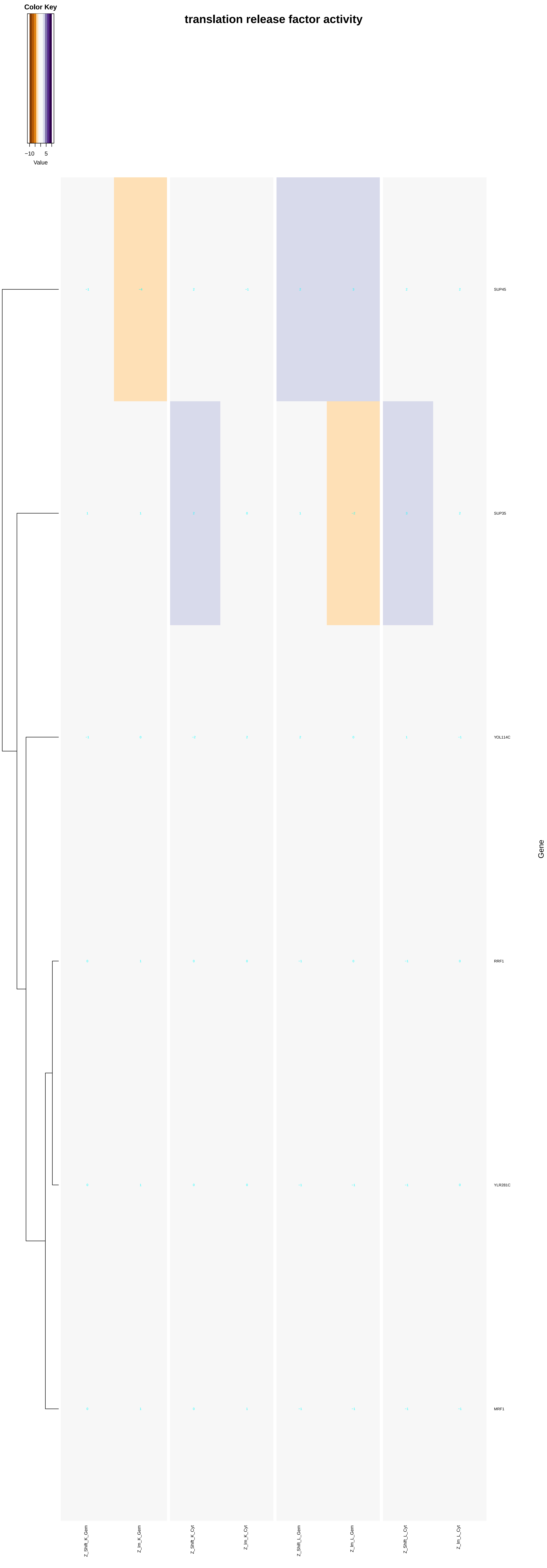

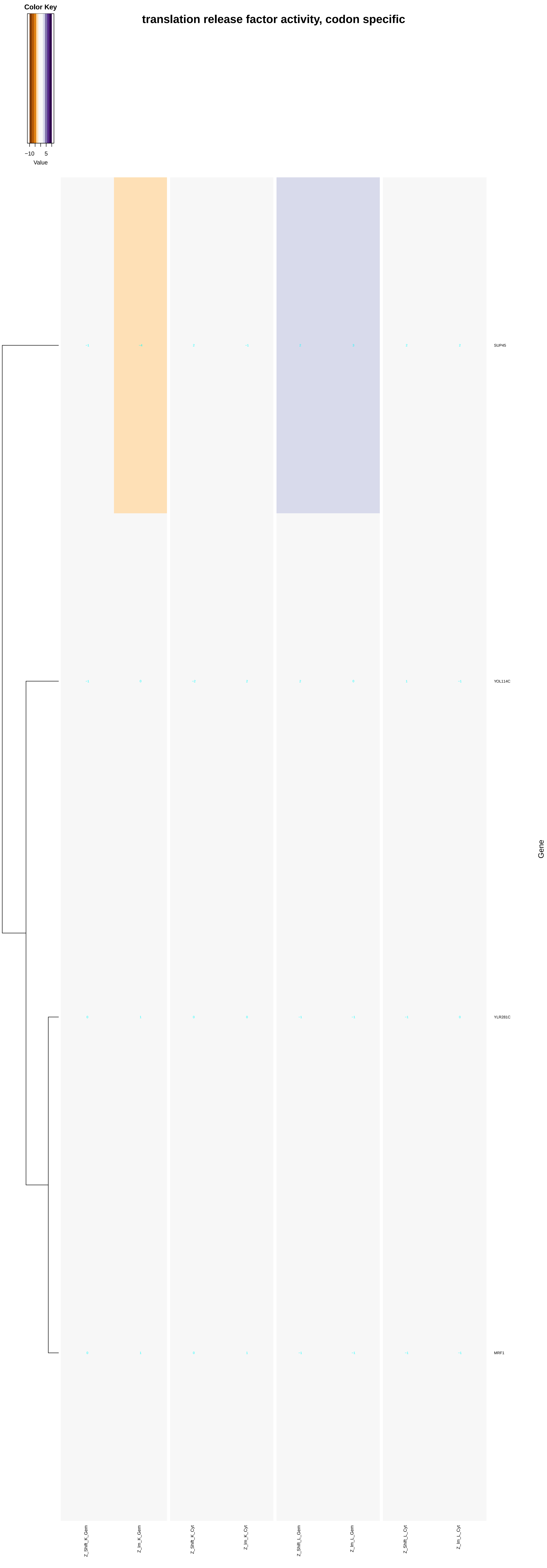
translation release factor activity, codon specific
Color Key
−10
5
Value
SUP45
YOL114C
YLR281C
MRF1
Gene
Z_Shift_K_Gem
Z_Im_K_Gem
Z_Shift_K_Cyt
Z_Im_K_Cyt
Z_Shift_L_Gem
Z_Im_L_Gem
Z_Shift_L_Cyt
Z_Im_L_Cyt
